# 

**Published:** 1904-07

**Authors:** 


July, 1904. Vol. XXV. No. 7

LIBRARY INDEXED

### THE INTERNATIONAL DENTAL JOURNAL.

JAMES TRUMAN, D.D.S., Editor,
PHILADELPHIA.

*COLLABORATORS:*

WILLIAM H. POTTER, A.B., D.M.D., BOSTON.
CHARLES P. BRIGGS, A.B., M.D., D.M.D., BOSTON.

### Summary of Contents.

(*For details, see p. 2.*)

ORIGINAL COMMUNICATIONS.

REPORTS OF SOCIETY MEETINGS.

BIBLIOGRAPHY.

REVIEWS OF DENTAL LITERATURE.

EDITORIAL.

OBITUARY.

CURRENT NEWS.

MISCELLANY.

LIBRARY
SURGEON GENERAL'S OFFICE
JUL. 6 1904

*$2.50 a Year, in Advance. Single Copies, 25 Cents.*

PUBLISHED MONTHLY BY

The International Dental Publication Company,

227-231 SOUTH SIXTH ST., PHILADELPHIA.

EDITORIAL OFFICE, 4505 CHESTER AVENUE, PHILADELPHIA, PA.

Printed by J. B. Lippincott Company, Philadelphia.

Entered at the Post-Office at Philadelphia, and admitted for transmission through the mails at second-class rates.

